# Functional assessment of older patients in the emergency department: comparison between standard instruments, medical records and physicians' perceptions

**DOI:** 10.1186/1471-2318-6-13

**Published:** 2006-09-04

**Authors:** Alejandro Rodríguez-Molinero, María López-Diéguez, Ana I Tabuenca, Juan J de la Cruz, José R Banegas

**Affiliations:** 1 Department of Preventive Medicine and Public Health. Universidad Autónoma de Madrid, Madrid, Spain

## Abstract

**Background:**

We evaluated the accuracy of physician recognition of functional status impairment in older emergency departments (ED) patients. In particular, we evaluated the accuracy of medical records (a comparison of the information in the medical record with the functional status based on proxy interviews), and the accuracy of physician knowledge (a comparison of the information obtained from the responsible physician with the functional status based on proxy interviews).

**Methods:**

Cross-sectional study on 101 frail older patients selected at random from among those attending ED, their ED physicians, and respondents. The study was conducted at ED in four general university teaching hospitals in a city, from July through November 2003. Functional data shown on patients' medical records were compared against functional data obtained from respondents (family members), using Kendall's Tau-b statistic. In addition patients' Katz Indices (which assesses six basic activities of daily living – basic ADL) based on interviews with ED physicians were compared against those obtained from respondents, using the coefficient of concordance weighted kappa (κ). Each patient and his respondent were paired with a single physician.

**Results:**

The correlation between information on dependence for basic ADL obtained from medical records and that furnished by respondents, was 0.41 (95% CI 0.27–0.55). Concordance between the respective Katz Indices obtained from physicians and respondents was 0.47 (95% CI 0.38–0.57).

**Conclusion:**

Older subjects' functional status is not properly assessed by emergency department physicians.

## Background

Emergency departments (ED) are used more frequently by older people than by the general population [1] and are not adequately prepared to attend to senior citizens' needs, which differ from those of the younger population [2].

Comprehensive geriatric assessment detects problems that traditional medical records tend to overlook [3,4]. One such problem is functional impairment and the dependence that ensues, a factor closely related to mortality among older people [5,6].

During older patients' visits to the emergency department, geriatric problems or risk factors for developing functional disability might be detected [7-10], and short-form assessment instruments requiring little implementation time have in fact been proposed for this purpose [11]. However, emergency department patients are known to be seldom questioned about their selfcare ability [12]. Consequently, functional dependence is often underdetected, poorly documented and inadequately addressed during encounters with older patients in an emergency setting [9-13].

Furthermore, scarcely any attention has been paid to the reliability and validity of the physician's judgment on assessing older patients' functional status in emergency departments, or how often emergency department physicians detect more disability than patients really suffer (overdiagnosis). This information would be important, since proper knowledge of older patients' prior functional status, influences prognosis as well as diagnostic and therapeutic decision-making. Functional status is strongly related with mortality and hospital outcomes [5], it can influence important decisions such DNR (do not resuscitate) order or access to intensive care units [14-17]. Furthermore, some evidence has been provided suggesting that physicians' impression on patients functional ability, predict mortality even better than organ damage or severity of illness. [18,19] Indeed comprehensive functional assessment in the emergency department has been shown to help decision-making with respect to discharge outcomes and planning of the healthcare needs of older patients [20]. Thus, an early geriatric evaluation in the ED has been advised [9].

In addition, no study has sought to explore whether the annotation of functional data on emergency department medical records has a positive effect on physicians' knowledge of this facet of their patients. It could be important to ascertain whether the emergency department medical record is a valid instrument for improving the transmition of functional status information through different doctors who take care of the same patient in the ED.

To assess the quality of the information collected by physicians, or documented on the emergency department medical record, it is necessary to use standardized information obtained from proxy respondents (generally a relative) as the reference standard, since it is not usually feasible for this to be directly obtained from older patients in an emergency situation [21]. Indeed, the proxy respondents of older patients have been shown to be a reasonable source of information on activities of daily living in cases where direct data-collection from patients themselves is not possible [22].

We evaluated the accuracy of physician recognition of ADL impairment in older ED patients. In particular, we evaluated the accuracy of medical records (a comparison of the information in the medical record with the functional status based on proxy interviews) and, in addition, we evaluated the accuracy of physician knowledge (a comparison of the information obtained from the responsible physician with the functional status based on proxy interviews).

Possible findings from such an analysis include detecting the possible presence of over- and under-diagnosis of functional status impairment. Finally, we measured the prevalence with which functional status was noted on ED medical records, and examined whether such annotation was associated with physician's gaining more accurate knowledge of their patients' functional status.

## Methods

### Study population

This study was conducted at four, general, university teaching hospitals in a city, from July through November 2003. These health centers are major hospitals in the city catchment's area, covering a reference population of 700,000. An average of 120000 patients attend each ED every year. Approximately 30% of these patients are 65 years old or older. Overall a 10% of the patients are finally admitted, however the observation areas of the emergency department where this study was conducted have a higher admission rate (up to 50%).

Five pre-trained clinician-researchers selected subjects from among frail older patients attending the respective hospital emergency departments during this period. All these patients were placed in observation areas, and therefore mild disease was previously excluded by triage physicians. Patients who attended the emergency department on each day of the study were numbered and afterwards a total of 106 subjects were selected, using a random numbers table. A frail older subject was defined as any person aged 80 years and older or, alternatively, any person aged 65 years and older suffering from two or more chronic diseases. Informed consent was given by all but five participants who declined to participate, therefore a total of 101 patients were finally included. Our institution exempted this study from formal review.

The clinician-researchers required the participation of all emergency department physicians who were directly responsible for patients, had never before participated in this study, and were not first-year medical residents, thereby making a total of 101 different emergency department physicians, one for each of the 101 patients. In cases where there were several physicians meeting these requirements for the same patient, the highest-ranking physician was chosen. Only one physician refused to participate: consequently, another available physician who was of equal rank and also carried responsibility was therefore selected in his stead.

After selecting a patient and a physician, the clinician-researchers registered the functional data furnished by the physician, then took note of the data standing on the medical record, and lastly, contacted respondents and examined the patient.

### Data-collection

"Baseline" functional status was defined as that predating the process or disorder for which patients had been attended at the emergency department.

Using questionnaires, the clinician-researchers recorded emergency department physicians' impressions of their patients' baseline functional status, by administering two standard instruments, namely: the Katz Index [23], to assess independence in six basic activities of daily living (basic ADL) (bathing, dressing, toileting, transfer from bed to chair, continence, and feeding); and the walking section of Barthel's Index [24]. For each of the activities included in these instruments, physicians' replies were categorized as dependent or independent in accordance with the pertinent guidelines [23,24]. The questionnaire was completed only after the emergency department physician had become familiar with the case of the patient that he/she attended and taken the pertinent clinical decisions. That is to say, while the physician, as the person responsible for the patient, had previously had access to the patient's medical record, clinical examination or information from other physicians, and had already taken clinical decisions, he/she was not allowed to consult any source of information at the time of completing the questionnaire.

Subsequently, the clinician-researchers reviewed the information available on the medical record of patients attended at emergency departments, noting down the current disease or, in default thereof, the reason for the medical visit, personal history and data on baseline functional status (measuring instruments, overall basic ADL, individualized basic ADL) and cognitive impairment (Pfeiffer's test administered to the patient by the clinician-researcher). Most medical records were drawn up by emergency department physicians in their first year of training.

Lastly, the clinician-researchers administered the Katz Index for six basic ADL and the walking section of Barthel's Index to respondents who claimed to be aware of patients' prior functional status (i.e., all the respondents). Replies were then categorized as "dependent" or "independent" for each of the "items". Missing data from respondents were partially recovered by subsequent telephone interview.

### Statistical analysis

For analysis purposes, all patients who agreed to participate in the study were included (n = 101), plus all physicians and respondents to whom the questionnaire had been supplied, regardless of whether or not they had completed it (n = 101). In all, 90 physicians and 90 respondents (90 pairs) furnished complete data on the patients.

The prevalence (and its 95% confidence interval) with which patient function data was present on the medical records kept by emergency departments, was calculated. Functional data shown on patients' medical records were then compared against functional data obtained from respondents, using Kendall's Tau-b statistic (n = 93) [25].

Furthermore, patients' Katz Indices based on interviews with emergency department physicians were compared against those obtained from respondents, using the coefficient of concordance weighted kappa (κ) (n = 90), with more weight being allocated to disagreements between categories or degrees of independence which were less extreme (i.e., deemed less serious) than to those which were more extreme [25].

The κ statistic was used to estimate the agreement between physicians and respondents for each basic ADL [10,26]. The κ index was also used to compare the Barthel Index question on walking administered to physicians and respondents. Moreover, weighted κ was obtained for Katz's physicians vs. Katz's respondents according to the number of basic ADL mentioned on the medical record (0, 1, and ≥ 2). In view of the small sample size available, figures were broken down into no more than three categories of basic ADL. A test for lineal trend was performed on the κ observed for these three basic ADL categories [27].

Lastly, the validity of physicians' opinions (sensitivity, specificity, and positive and negative predictive values) was calculated, using patient information obtained from the respondent as the reference standard. 95% confidence intervals were computed for all indices [27].

## Results

### Baseline characteristics of patients

The baseline characteristics of the study population are summarized in Table 1. These data were drawn from information furnished by patients' respondents, except for the cognitive test, which was measured directly by the clinician-researchers.

**Table 1:** Sociodemographic, cognitive and functional characteristics of the patient sample (n = 101)

**Gender**	n (%)
Male	60 (61.2%)
Female	38 (38.8%)
**Age (Mean ± SD)**	81.7 ± 7.3
**Educational level**	
No formal education	46 (47.4%)
Elementary education	47 (48.5%)
University education	4 (4.1%)
**Marital status**	
Single	5 (5.1%)
Married	46 (46.9%)
Widowed	46 (46.9%)
Separated/divorced	1 (1.0%)
**Living**	
Alone	7 (7.2%)
In a residence	3 (3.1%)
With spouse	35 (36.1%)
With children	34 (35.1%)
With spouse and children	8 (8.2%)
Other	10 (10.3%)
Pfeiffer's test*	
Positive	43 (48.3%)
Negative	46 (51,7%)
**Katz**	n (%)
Totally independent	34 (35.4%)
Totally dependent	8 (8.3%)
Bathing	
Independent	55 (55.6%)
Dependent	44 (44.4%)
Dressing	
Independent	75 (75.8%)
Dependent	24 (24.2%)
Toileting	
Independent	71 (71.7%)
Dependent	28 (28.3%)
Transfers from bed to chair	
Independent	72 (73.5%)
Dependent	26 (26.5%)
Continence	
Independent	44 (44.9%)
Dependent	54 (55.1%)
Feeding	
Independent	90 (91.8%)
Dependent	8 (8.2%)
Walking	
Independent	59 (62.8%)
Dependent	35 (37.2%)

*Cognitive impairment screening test.

The emergency department physicians had a mean age of 30.9 years (± 5.2) and were predominantly female (66.7%), with 42.6% being internal-medicine specialists, 27.7% family and community medicine specialists, and the rest other types of specialists. Hospital staff physicians, who have more than 5 years of clinical experience, accounted for the 35.4% of the total, and the remainder were residents and fellows who had two to five years of clinical experience.

Insofar as the respondents were concerned, 66.7% were women and the mean age was 56.1 years (± 12.6); 66.3% were offspring, 19.4% spouses, and 13.3% other relatives of patients, while 1% had no family relationship with the patient.

### Medical records

No reference was made to patients' functional ability on 75 emergency department medical records (75%). We searched for specific mention of continence, bathing, toileting, transfer from bed to chair, dressing, feeding and walking: only 16 (16%) records referred to one and 9 (9%) to two or more of these basic ADL. Both disabled and independent patients had an important lack of ADLs information on their medical records. (Table 2)

**Table 2:** Number of ADLs mentioned on clinical history, according to number of disabilities of the patients.

	Patients disabilities				
	5–6 ADL disability	3–4 ADL disability	1–2 ADL disability	No ADL disability	All patients
No mention (n,%)	10 (**58**%)	6 (**60**%)	28 (**80**%)	28 (**82**%)	72 (75%)
Bathing mentioned (n)	1	0	1	1	3
Dressing mentioned (n)	1	0	0	1	2
Toileting mentioned (n)	0	1	1	1	3
Transferring mentioned (n)	2	0	1	1	4
Continence mentioned (n)	4	3	3	5	15
Eating mentioned (n)	1	0	0	0	1
Walking mentioned (n)	3	2	5	3	13
N	17	10	35	34	96

The basic ADL most frequently noted on medical records overall, were walking (12.9%) and continence (15.8%). In no medical record was there any element of a standard measure to stratify degree of dependence or independence for basic ADL.

The correlation (Tau-b statistic) between information on dependence for basic ADL obtained from medical records and that furnished by respondents, was 0.41 (95% CI 0.27–0.55).

### Medical judgment

Concordance between the respective Katz Indices obtained from physicians and respondents was 0.47 (95% CI 0.38–0.57). Concordance for the different basic ADL, along with the sensitivity, specificity and predictive values of medical judgment for the purpose of detecting functional disability for these basic ADL, are shown in Table 3.

**Table 3:** Concordance and validity indexes between patients' baseline functional data obtained from physicians and those obtained from respondents, according to each ADL items.

	Kappa coefficient (95% CI)	Sensitivity (%) (95% CI)	Specificity (%) (95% CI)	Positive predictive value (%) (95% CI)	Negative predictive value (%) (95% CI)
Katz (n = 90)	0.47 (0.38–0.57)				
Bathing (n = 97)	0.34 (0.14–0.54)	69.8 (56.0–83.5)	64.8 (52.1–77.6)	61.2 (47.6–74.9)	72.9 (60.3–85.5)
Dressing (n = 96)	0.42 (0.24–0.60)	87.5 (74.3–100)	66.7 (55.8–77.6)	46.7 (32.1–61.2)	94.1 (87.7–100)
Toileting (n = 96)	0.39 (0.20–0.58)	71.4 (54.7–88.2)	72.1 (61.4–82.7)	51.3 (35.6–67.0)	86.0 (77.0–95.0)
Transfer from bed to chair (n = 93)	0.32 (0.12–0.52)	64.0 (45.2–82.8)	72.1 (61.4–82.7)	45.7 (29.2–62.2)	84.5 (75.2–93.8)
Continence (n = 94)	0.28 (0.08–0.48)	61.5 (48.3–74.8)	66.7 (52.4–80.9)	69.6 (56.3–82.9)	58.3 (44.4–72.3)
Feeding (n = 95)	0.29 (0.10–0.48)	50.0 (15.4–84.7)	88.5 (81.8–95.2)	28.6 (4.9–52.2)	95.1 (90.3–99.8)
Walking (n = 92)	0.34 (0.14–0.55)	64.7 (48.6–80.8)	70.7 (59.0–82.4)	56.4 (40.9–72.0)	77.4 (66.1–88.6)

**Figure 1:**
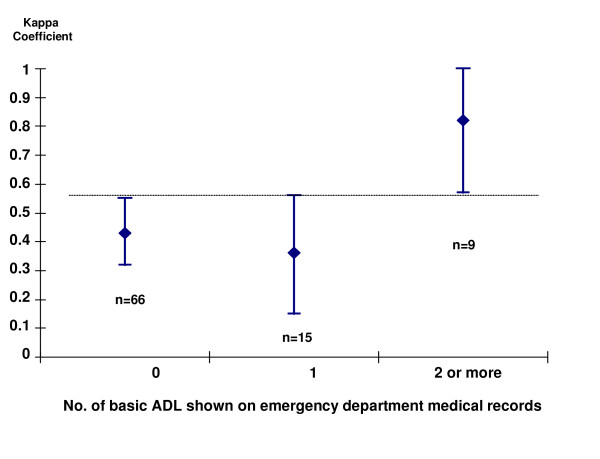
Concordance (κ coefficient) between patients' functional status (Katz Index) reported by physicians and that reported by respondents, according to the number of basic activities of daily living (basic ADL) 'mentioned in the medical record. Bars correspond to the 95% confidence intervals (95% CI) of κ. The broken line highlights the fact that there is no overlap between the 95% CI for 0 and for 2 or more basic ADL. The sample sizes (n) for each basic ADL subgroup are shown beneath the bars.

Among the subgroup of patients whose emergency department medical records made no mention of basic ADL, concordance between physicians' knowledge and the information furnished by relatives was only moderate (κ = 0.43, 95% CI 0.32–0.55). In cases where one basic ADL was specifically mentioned, concordance continued to be moderate (κ = 0.36, 95% CI 0.15–0.56), but where two or more basic ADL were mentioned, concordance rose to 0.82 (95% CI 0.57–1.00) (*P *for trend < 0.001) (Figure 1).

## Discussion

### Main findings

In this sample of emergency department medical records, functional data are poorly documented (altogether lacking in 75% of). Moreover, this information on functional ability is not structured when reflected on the medical record, with only 9% of all records independently mentioning at least two basic ADL. The correlation between the functional data shown on the medical record and patients' Katz Index was only moderate (Kendall's Tau-b 0.41).

Agreement between physicians and relatives as to functional status (κ = 0.47) would seem scant in view of the importance of the variable, it being desirable for physicians to have a better knowledge of their patients' functional status. However, the small sample size of this study means that the degree of agreement has a relatively wide confidence interval (0.38–0.57) and must thus be interpreted with caution.

As far as clinical judgment accuracy is concern, our data indicate that the sensitivity of clinical judgment is acceptable for the purpose of detecting dressing- or bathing-related dependence. However, many patients with disability for other basic ADL, such as bladder and bowel control, feeding or transfers from bed to chair, are not diagnosed as dependent for such activities by physicians, and, as a result, these problems cannot be expected to be properly tackled.

In general, the specificity of medical diagnosis is not high either, indicating that a good few patients who are not disabled for the purpose of certain basic ADL are nevertheless being included in the group of dependents. The magnitude of this fact is better appreciated in terms of the positive predictive value. It is noteworthy that physicians err more than 50% of the time that they diagnose patients as dependent for dressing, toileting, transfers from bed to chair, or feeding. That is to say, approximately half of all disability judgments made by the physician are incorrect, which translates into overdiagnosis of disability, something that can negatively influence the therapeutic plans for such patients.

As a sencondary finding, our data show that the degree of agreement between clinical judgment and relatives is notably increased by at least two basic ADL being documented on the medical record. Where two basic ADL were mentioned, κ agreement was 0.59 (95% CI 0.34–0.84) (data not shown). Yet, where ≥ 2 basic ADL were recorded, the κ index was very good indeed (0.82), and this improvement (as compared with κ for 0 basic ADL) is not explained by the width of the confidence intervals, since these κ intervals for zero (0.32–0.55) and ≥ 2 basic ADL (0.57–1.00) did not overlap. Moreover, although this study was not specifically designed to examine differences in medical knowledge of patients' functional status according to express mention of basic ADL on the medical record (this was a secondary objective), even with only 9 subjects in the subgroup with ≥ 2 basic ADL, a clinically relevant relationship (κ = 0.82) was detected. This improvement in the κ index seems plausible. If the record is more detailed, physicians form a more accurate picture of patient's baseline status and tend to include fewer healthy persons in the disabled group, thereby reducing the dangerous overdiagnosis of disability.

### Strengths and weaknesses of the study

As a result of the interview carryout by the researchers, doctors included may learn somewhat about the importance of the functional status of their patients, therefore collecting more accurate functional information from patients attended soon after. That is why we designed our study selecting 101 doctor-patient pairs, which means that doctors were interviewed just once, concerning a single patient. We consider this approach more trustworthy to obtain a reliable picture of functional knowledge in EDs.

One limitation of this study is its small sample size, which may pose problems of precision (discussed above) and generalizability. The sample size employed is in part a consequence of the difficulty of enrolling 101 different emergency department physicians, i.e., physicians who had not previously participated in this study. To minimize the increase in prevalence of functional data reflected on the medical record as the study progressed (contamination of the sample due to the action of clinician-researchers in the emergency department), the study was extended to four hospitals, and data-collection was spread over time. Moreover, the lack of an emergency medicine specialty in Spain could affect the generalizability of our results to other countries whose ED physicians are specifically trained.

Furthermore, this study was cross-sectional in design, which restricts the drawing of causal conclusions. Hence, just as greater detail on basic ADL in the medical record could have a positive effect on emergency department physicians' knowledge of patients' functional status, so might it be theoretically possible for better knowledge on physicians' part to have an influence on more detailed annotation of the medical record. Yet, it is unlikely that knowledge held by the physician responsible for the patient could have any influence on medical records that had previously been drawn up -in most cases- by another physician. For this precise reason, it is interesting to underscore the fact that, in general, detailed annotation of medical records by certain physicians could serve to enhance the knowledge enjoyed by other physicians, nevertheless as this study has not been specifically designed to address this point, further investigation may be needed to confirm this finding.

A further limitation of the study is that it was not feasible for different clinician-researchers to be used to collect information separately from respondents, physicians and medical records. The fact that clinician-researchers were not blind to the answers of the respective participants (physicians and respondents) was a limitation that was sought to minimize by ensuring a strict data-collection order, i.e., only after gathering information from physicians and medical records was the Katz Index administered to relatives, thereby preventing any influence being exerted by the latter on physician-based data and records.

Lastly, patients who attended the emergency department without a respondent who could serve as the reference standard were excluded from this study. Consequently, we did not study physicians' knowledge of the functional status of patients who were not accompanied by a respondent, but it is to be expected that such knowledge would be less that that shown by the results of this study, particularly in the case of patients with cognitive impairment.

### Consistence with other studies

A lack of functional data in the medical records has been previously observed by other authors. Bogardus and colleagues obtained a figure of 61%–98% for medical records that did not document functional data [28]. Consistent with our findings, Currie *et al *also observed that in emergency departments, medical records held scant information on functional and social status [29].

Some authors have observed that, compared with patient self-report data, the sensitivity and specificity of functional data registered in the medical records of hospitalized patients are low (48%–68% and 64%–82%, respectively) [30]. We were unable to find similar studies with respect to emergency departments.

Insofar as the opinion of physicians is concerned, there are hardly any references in the literature to the reliability and validity of medical judgment when it comes to assessing patients' functional status in the emergency department, though this aspect has been studied by some authors in the context of ambulatory patients. Incontinence measured in hospitalized patients is detected by general practitioners and nurses in only 65% of cases [31]. Similarly, physicians' lack of recognition and overdiagnosis of disability for transfers from bed to chair and walking among ambulatory patients has been highlighted [4].

According to previous experiences in other settings, our study shows a limited functional data information in the emergency department clinical records. Interestingly, our study also demonstrates a lack of knowledge of this information by emergency department physicians, which in our opinion is a novel finding.

### Meaning of the study and implications for clinicians or policymakers

The fact that emergency department medical records furnish inadequate information on patients' functional ability renders it especially important to assess physicians' ability to detect both disability-free patients and those with major disability. The presence or absence of major disability influences decision-making, and poor knowledge of functional status can therefore lead to inappropriate decisions.

The results highlight a lack of an information that may be essential in the management of elderly patients at the ED. Therefore we consider a bigger effort may be done in the EDs to assess and document patients functional status. The use of relatively simple instruments to assess the functional status of the older in emergency departments would therefore appear reasonable [32]. These instruments contribute to reducing the rate of functional impairment on discharge [5,10] and, in our opinion, may well help improve emergency departments decision-making, though further research in this area is called for [33].

As a practical implication of this study, our results suggest that the accuracy of medical judgment on the functional status of older patients in the emergency department could improve, no matter how little the information on basic ADL available to them on the medical record were to increase.

### Unanswered questions and future research

An association between clinical documentation and physician knowledge has been suggested in this study, but the study was not primary designed to clarify this point. We consider further research is necessary in order to address the importance of clinical record as a valid instrument to transfer functional information through different doctors, therefore reducing the inaccuracy of functional knowledge we have found in the ED setting.

## Conclusion

In conclusion, the functional status of frail older patients is inadequately reflected in emergency department medical records. Senior citizens' functional ability is in general not properly detected by emergency department physicians, and disability tends to be overdiagnosed. Finally, emergency department physicians register better knowledge of functional status when this has been detailed in a structured manner on the medical record. The mere annotation of at least two basic ADL on emergency department medical records is associated with physicians' better knowledge of emergency patients' basal functional status. This in turn may contribute to improving clinical decision-making in the case of frail patients attended at hospital emergency departments.

## Competing interests

The author(s) declare that they have no competing interests.

## Authors' contributions

ARM and JRB conceived the study. All authors (ARM, MLD, AIT, JJC and JRB) participated in study design and data analysis. AIT and JJC did statistical analyses. All authors participated in interpretation of data and preparation of the manuscript. All authors read and approved the final manuscript.

## Pre-publication history

The pre-publication history for this paper can be accessed here:


